# Rb Diffusion and Oxide Removal at the RbF-Treated Ga_2_O_3_/Cu(In,Ga)Se_2_ Interface in Thin-Film Solar Cells

**DOI:** 10.1021/acsami.3c11165

**Published:** 2023-11-01

**Authors:** Elizaveta Pyatenko, Dirk Hauschild, Vladyslav Mikhnych, Raju Edla, Ralph Steininger, Dimitrios Hariskos, Wolfram Witte, Michael Powalla, Clemens Heske, Lothar Weinhardt

**Affiliations:** † Laboratory for Applications of Synchrotron Radiation (LAS), Karlsruhe Institute of Technology (KIT), Kaiserstraße 12, Karlsruhe 76131, Germany; ‡ Institute for Photon Science and Synchrotron Radiation (IPS), Karlsruhe Institute of Technology (KIT), Hermann-v.-Helmholtz-Platz 1, Eggenstein-Leopoldshafen 76344, Germany; § Institute for Chemical Technology and Polymer Chemistry (ITCP), Karlsruhe Institute of Technology (KIT), Engesserstraße 18/20, Karlsruhe 76128, Germany; ∥ Department of Chemistry and Biochemistry, University of Nevada, Las Vegas (UNLV), 4505 Maryland Parkway, Las Vegas, Nevada 89154-4003, United States; ⊥ Zentrum für Sonnenenergie- und Wasserstoff-Forschung Baden-Württemberg (ZSW), Meitnerstraße 1, Stuttgart 70563, Germany

**Keywords:** Cu(In,Ga)Se_2_ thin-film solar cells, RbF postdeposition treatment, ammonia-based rinse, photoelectron spectroscopy, HAXPES, chemical structure, surface oxides, gallium oxide buffer layer

## Abstract

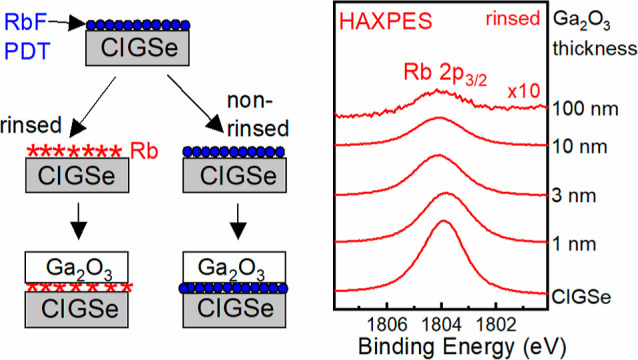

We report on the chemical structure of Cu(In,Ga)Se_2_ (CIGSe) thin-film solar cell absorber surfaces and their interface with a sputter-deposited Ga_2_O_3_ buffer. The CIGSe samples were exposed to a RbF postdeposition treatment and an ammonia-based rinsing step, as used in corresponding thin-film solar cells. For a detailed chemical analysis of the impact of these treatments, we employed laboratory-based X-ray photoelectron spectroscopy, X-ray-excited Auger electron spectroscopy, and synchrotron-based hard X-ray photoelectron spectroscopy. On the RbF-treated surface, we find both Rb and F, which are then partly (Rb) and completely (F) removed by the rinse. The rinse also removes Ga–F, Ga–O, and In–O surface bonds and reduces the Ga/(Ga + In) ratio at the CIGSe absorber surface. After Ga_2_O_3_ deposition, we identify the formation of In oxides and the diffusion of Rb and small amounts of F into/onto the Ga_2_O_3_ buffer layer but no indication of the formation of hydroxides.

## Introduction

The development of alkali-fluoride postdeposition treatments (PDTs) has led to a significant efficiency increase for Cu(In,Ga)Se_2_ (CIGSe) and Cu(In,Ga)(S,Se)_2_ solar cells, first to 20.4% using a KF-PDT,^1^ then to 22.6% with RbF-PDT,^2^ and to 23.35% by using CsF-PDT.^3^ PDTs are most often followed by a rinsing step (e.g., with H_2_O or an NH_3_ solution) and/or a chemical bath deposition (CBD) step used for the subsequent deposition of a buffer layer, which removes excess alkali fluoride.^4−6^ In general, rinsing steps of CIGSe absorber surfaces have been employed ever since Na residue (from the soda-lime glass substrate) was observed on CIGSe surfaces,^7−11^ leading to a reduction or even complete removal of alkali metals^12^ and, in some cases, even oxides^13,14^ from the absorber surface.

Chemical-bath-deposited CdS (CBD-CdS) is generally used as the buffer layer in high-efficiency CIGSe-based thin-film solar cells,^2,15−17^ while the current world records for mini modules and lab-scale cells have already utilized sputtered Zn(O,S)^18^ or CBD-Zn(O,S,OH)^3^ buffers, respectively. Avoiding the use of CBD-CdS is motivated by reducing the environmental impact of the production process,^19,20^ reducing the parasitic absorption,^21−23^ and developing a “dry” process that does not interrupt a vacuum-based process chain.^20,24^

Thus, alternative buffer layer materials, including In_*x*_S_*y*_,^24−28^ Zn(O,S,OH),^3,28,29^ (Zn,Mg)O,^30−32^ Zn_1–*x*_Sn_*x*_O_*y*_,^33,34^ HfO_*x*_,^35^ Al_2_O_3_,^28,36,37^ Ga_2_O_3_,^23,24,38^ Sn_1–*x*_Ga_*x*_O_*y*_,^22^ (In,Ga)_2_O_3_,^23^ and (Al,Ga)_2_O_3_,^23^ have been investigated. A particularly promising candidate among the alternative buffer layers is the wide band gap transparent oxide Ga_2_O_3_, with an optical bulk band gap between 4.4 and 4.9 eV.^21,24,39−43^ Previously, a thin Ga_2_O_3_ layer was also applied as a passivation layer at the CdS/CIGSe interface (highest efficiencies were achieved with a 0.6 nm passivation layer). This improved the open-circuit voltage (*V*_oc_), the short-circuit current density (*J*_sc_), and the fill factor (FF), resulting in an efficiency increase of 2.6% (absolute) as compared to the reference cells without the Ga_2_O_3_ passivation layer.^21^ Furthermore, amorphous (In_1–*x*_Ga_*x*_)_2_O_3_ buffer layers (with *x* ranging from 0.6 to 1) were investigated, achieving efficiencies (∼15.8%) close to those of reference cells with CdS buffers (∼17.2%) for *x* = 1 (i.e., pure Ga_2_O_3_).^23^ The highest efficiencies for these cells were achieved for buffer layer thicknesses of 60 nm.

In the first trial, ZSW used sputter-deposited gallium oxide as a buffer layer for CIGSe absorbers, reaching efficiencies up to ∼13.7% (compared to ∼17.6% with a CdS reference buffer).^24^ Furthermore, replacing i-ZnO in a ZnO/Al/i-ZnO/CdS/CIGSe structure by sputtered Ga_2_O_3_ resulted in cell efficiencies of 20.2% (compared to 20.4% for the reference cell).^44^ The absorbers were exposed to RbF-PDT as in the CdS-based ZSW process that has led to efficiencies above 22%.^2^ Additionally, a rinse in a 1.5 M NH_3_ solution to remove surplus RbF as well as a temperature optimization series for the buffer deposition were performed. Due to the wider band gap of Ga_2_O_3_, an increase in *J*_sc_ compared to reference cells with CdS was observed.^24,44^

In the current work, we present a detailed investigation of the chemical structure of the RbF-PDT CIGSe absorber surface, the sputter-deposited Ga_2_O_3_ buffer layer, and the Ga_2_O_3_/CIGSe interface. Particular focus is placed on the investigation of the ammonia-based rinsing step and its impact on the chemical structure of the absorber surface and the buffer/absorber interface, which is substantially more complex than a simple removal of RbF. For this purpose, samples were studied by laboratory-based X-ray photoelectron (XPS) and X-ray-excited Auger electron spectroscopy (XAES) as well as synchrotron-based hard X-ray photoelectron spectroscopy (HAXPES). Findings in this work can be correlated to electrical device parameters as reported in refs (24) and (44).

## Experimental Section

The investigated samples were prepared at ZSW; the CIGSe absorbers were grown in a high-vacuum chamber by coevaporating Cu, In, Ga, and Se in an in-line multistage process onto a molybdenum/soda-lime glass substrate.^45^ The bulk [Ga]/([Ga] + [In]) (GGI) ratio was determined by X-ray fluorescence (XRF) measurements as 0.27, and the integral Cu content was found to be 21.3 at %.

The RbF PDT was applied in the same high-vacuum chamber without breaking the vacuum after the CIGSe process. Two different sample sets were prepared: the first set of absorbers was rinsed in 1.5 M NH_3_ solution for 30 s (referred to as “rinsed” in the following), while the second set was not rinsed (“non-rinsed”). The ammonia concentration for the rinsing solution, which is the same as the concentration used for the CBD CdS deposition, and the rinsing duration of 30 s were found to be sufficient for dissolving surplus RbF from the CIGSe surface.

Subsequently, Ga_2_O_3_ was deposited by radiofrequency (RF) magnetron sputtering from a ceramic target at a substrate temperature of 150 °C (details of the Ga_2_O_3_ sputter-deposition process can be found in refs (24) and (44)), generating films with thickness *d* of 1, 3, 10, and 100 nm. The thicknesses were estimated from sputter-deposition rates determined by optical transmittance measurements on thick Ga_2_O_3_ layers (*d* > 200 nm) deposited on 1 mm highly transparent quartz-glass substrates. Sister samples, processed to full solar cells (i.e., with an approximately 100 nm thick Ga_2_O_3_ buffer layer and a sputtered i-ZnO/ZnO/Al transparent front contact), showed maximum efficiencies of ∼5% for the non-rinsed and above 13% for the rinsed CIGSe absorbers, in comparison to above 16% for CdS-buffered reference devices.^44^ Efficiencies were measured without antireflective coating on solar cells with a total area of 0.5 cm^2^ with Ni/Al/Ni grids on top.

After preparation at ZSW, the samples were briefly exposed to air, sealed in a dry nitrogen atmosphere, and transported to KIT. There, the sealed samples were unpacked and mounted in an Ar-filled glovebox at the Materials for Energy (MFE) laboratory. Without any air exposure, the mounted samples were transferred directly from the glovebox into the ultrahigh vacuum (UHV) system for XPS and XAES measurements. XPS measurements were performed with a Scienta Omicron Argus CU electron analyzer, a non-monochromatized DAR 450 Mg K_α_ X-ray source (Scienta Omicron), and a monochromatized MECS Al K_α_ X-ray source (SIGMA Surface Science). The base pressure in the XPS chamber was less than 2 × 10^–10^ mbar. After the initial XPS and XAES measurements, the samples were transferred to the X-SPEC beamline^46^ at the KIT Light Source. After a brief exposure to air (less than 30 s), HAXPES spectra were measured with a Phoibos 225 electron analyzer (SPECS) and a photon energy of 2.1 keV using the Si(111) reflection of the double-crystal monochromator. The base pressure in the HAXPES analysis chamber was less than 5 × 10^–10^ mbar.

To calibrate the XPS/XAES measurements, the most prominent photoemission and Auger peaks of sputter-cleaned Au, Ag, and Cu foils were used.^47,48^ The HAXPES binding energies were calibrated using the Au 4f_7/2_ peak^46,47^ of a reference Au foil.

## Results

**Figure 1:**
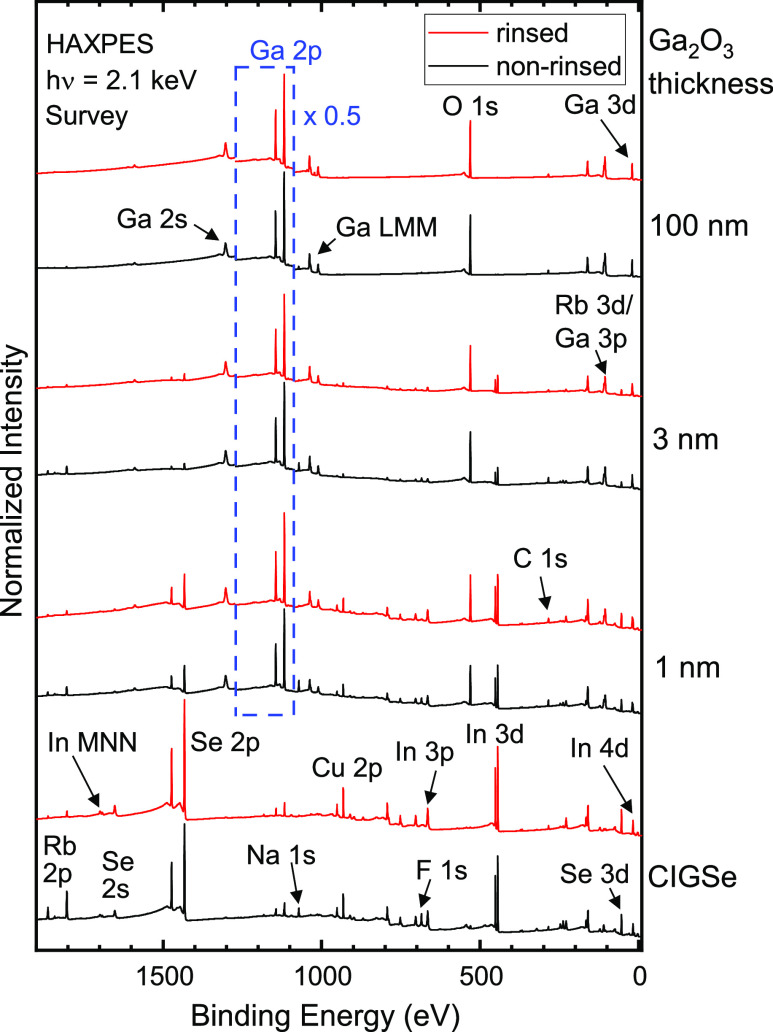
HAXPES survey spectra of the CIGSe absorber with RbF-PDT and the 1, 3, and 100 nm Ga_2_O_3_/CIGSe samples, measured at an excitation energy of 2.1 keV. The red and black spectra correspond to the rinsed and non-rinsed sample series, respectively. Spectra were normalized to their overall integral intensity. The Ga 2p_3/2_ signals of the Ga_2_O_3_ buffer layers are multiplied by a factor of 0.5 for better visibility (blue dashed box). Prominent photoemission peaks and Auger signals are labeled.

Figure 1 shows selected HAXPES (*h*ν_exc_ = 2.1 keV) survey spectra of the rinsed (red) and non-rinsed (black) absorbers, as well as the corresponding 1, 3, and 100 nm Ga_2_O_3_/CIGSe interface samples. Figure S1 shows the corresponding Mg K_α_ XPS survey spectra. For the CIGSe absorbers, the expected core levels and XAES lines of the absorber elements (Cu, In, Ga, and Se) are detected. Also, the Rb 2p lines (Figure 1) are clearly visible (*E*_B_ ∼ 1800 eV), demonstrating one strength of HAXPES to also detect such deeply bound core levels; in the case of Rb on CIGSe, uniquely identifying Rb with any other line is extremely challenging. With increasing buffer layer thickness, the Cu, Se, and In signals are increasingly attenuated by the Ga_2_O_3_ overlayer, while the Ga and O signals show a strong enhancement.

Due to the higher excitation energy used for HAXPES as compared to laboratory XPS, the kinetic energy of corresponding core-level peaks increases, and thus the HAXPES measurements are less surface-sensitive than the XPS measurements. For instance, the inelastic mean free path λ for the In 3d feature is 3.0 nm for HAXPES and 1.7 nm for XPS, respectively.

In the XPS data (Figure S1), all absorber-related lines are fully attenuated for the thickest buffer layer (100 nm) samples, while the HAXPES data show very small Cu 2p, In 3d, and Se 3d signals for the rinsed and non-rinsed sample sets (enabled by the excellent signal-to-noise ratio obtainable at the X-SPEC beamline). This could be due to regions with a lower buffer layer thickness (or even a not fully closed buffer layer), a diffusion of the absorber elements into (or onto) the buffer layer, or a combination thereof. Note that a cross-section image of the Ga_2_O_3_/CIGSe interface^44^ suggests that regions with lower buffer layer thickness are unlikely but cannot be entirely excluded for this sample series. In both absorber spectra, small C and O 1s signals are visible. For the non-rinsed absorber, strong Rb-, F-, and Na-related signals, the latter likely due to diffusion from the soda-lime glass,^9^ are also detected.

A comparison of the non-rinsed and rinsed CIGSe absorbers (Figure 1) shows that the CIGSe-related lines (e.g., Cu 2p, In 3d, and Se 3d) increase after the ammonia rinse by a factor of ∼1.3. In parallel, we find a strong decrease in the intensities of the Rb 2p, F 1s, Na 1s, and O 1s lines, while the C 1s intensity increases (by a factor of 1.2). O 1s and C 1s detailed spectra are shown in Figure S2, and Na 1s spectra are presented in Figure S3. We interpret these spectral changes as follows: the rinse removes excess material of RbF-PDT (i.e., Rb and F), Na, and surface oxides (as will be discussed below) from the absorber surface. The absorber-related signals (as well as tightly bound CIGSe surface species, e.g., carbon) then increase in intensity due to the reduced attenuation by this surface layer. The larger increase in the C 1s signal for the rinsed absorber may also be due to a more “reactive” surface after the rinse that is more susceptible to the adsorption of carbon-containing species during the subsequent sample handling.

**Figure 2:**
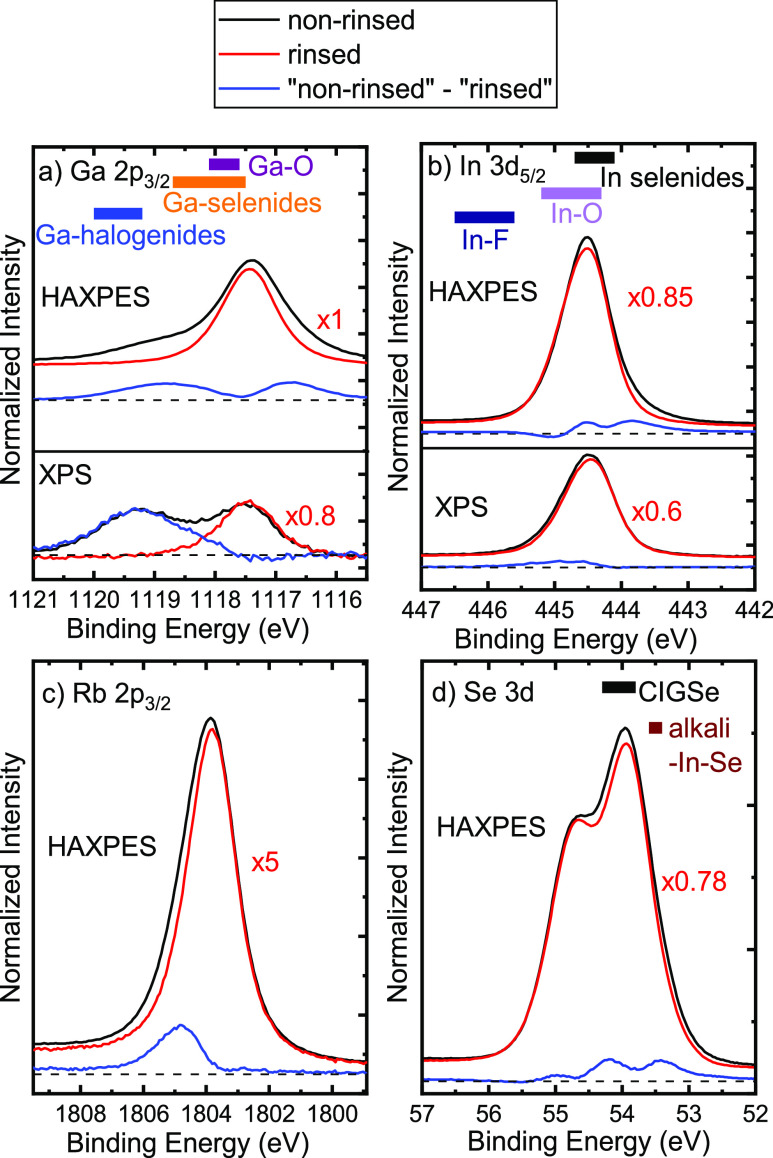
HAXPES (*h*ν = 2.1 keV) and monochromatized Al K_α_ XPS spectra of the (a) Ga 2p_3/2_, (b) In 3d_5/2_, (c) Rb 2p_3/2_, and (d) Se 3d regions. The black and red lines correspond to the non-rinsed and rinsed CIGSe absorbers, respectively. All spectra were normalized by the same factors as the survey spectra in Figure 1. In addition, the given multiplication factors were applied to the red spectra (rinsed) in the figure in order to maximize the contribution of the respective component. The blue lines then show the difference spectra between “non-rinsed” and “rinsed” surfaces. Colored bars show ranges of literature values of the binding energies for different compounds.^4,49,50^

To study the differences in the chemical environment at the surfaces of the non-rinsed and rinsed absorbers, we discuss the Ga 2p_3/2_, In 3d_5/2_, Rb 2p_3/2_, and Se 3d spectral regions in Figure 2.

In Figure 2a, prior to the rinse, we find a spectral component indicating Ga–F bonds (the blue box shows literature values for Ga in GaCl, GaBr_3_, and GaI_3_).^50^ After the rinse, this component disappears, as indicated by the difference spectrum (blue). This is in accordance with the F signal, which vanishes after the rinse (as discussed later in this paper). The Ga–F environment is much stronger in the more surface-sensitive XPS measurement, and the overall Ga 2p_3/2_ signal is reduced after the rinse, which we explain as follows: the RbF-PDT forms Ga–F bonds with Ga at the surface, and the rinse washes away F and (some of the) Ga, reducing the GGI ratio at the surface. This finding is supported by inductively coupled plasma–mass spectrometry, where Ga was found in the rinsing residue.^25^ This change in the Ga content at the surface might impact (reduce) the surface band gap and the performance of the cells.^51^

**Figure 3:**
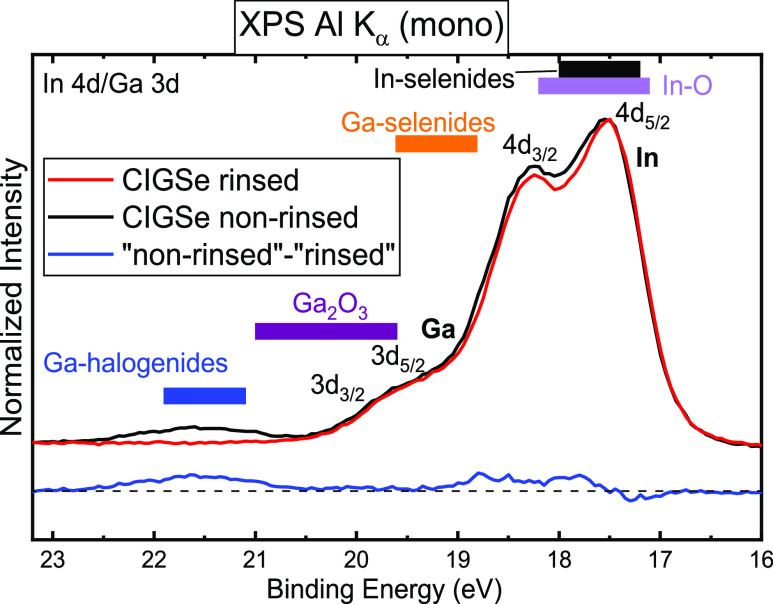
Monochromatized Al K_α_ XPS spectra of the In 4d/Ga 3d region. The black and red lines correspond to the non-rinsed and rinsed CIGSe absorbers, respectively. The spectra were normalized to their overall maximum. The blue line shows the difference spectrum between “non-rinsed” and “rinsed”. Colored bars show ranges of literature binding energies for different compounds.^49,50^

In Figure 2b, we see no indication of In–F bonds. However, the XPS spectrum shows a weak shoulder at ∼445.5 eV that is removed after the rinse, likely due to the reduction in In–O bonds. This observation will be discussed in conjunction with Figure 3.

There are additional shoulders at low binding energies for the HAXPES Ga 2p_3/2_ and In 3d_5/2_ spectra of the non-rinsed samples in Figure 2a,b, respectively. In the case of Ga 2p_3/2_ spectra, this additional component can be assigned to the Na KL_2,3_L_2,3_ Auger transition at ∼1112 eV,^49^ which is removed after the rinse. The low-binding energy shoulder of the In 3d_5/2_ spectra at ∼443.8 eV might be attributed to In in the Rb–In–Se environment present at the surface before the rinse, as we will discuss below.

Figure 2c shows that the Rb signal decreases by more than a factor of 5 after the rinse. In addition, the difference spectrum clearly highlights that there is an additional spectral component in the non-rinsed absorber at higher binding energies. We assign it to an Rb–F environment, which, similarly to Ga–F, is removed by the rinse as well. The main spectral component can be attributed to Rb at the CIGSe surface,^52,53^ while the Se 3d spectra in Figure 2d indicate only a small contribution from a Rb–In–Se environment before the rinse, indicated by a weak additional spectral component at low binding energies. The binding energy corresponds to that of an alkali–In–Se environment, as reported in ref (4). We observe a narrowing of the line after the rinse, which may indicate that there are several slightly different chemical environments present on the non-rinsed sample. In ref (4), a shift of ∼0.5 eV was observed for the In 3d_5/2_ peak between In in a CIGSe and In in an alkali–In–Se environment. The weak low-binding energy shoulder (443.8 eV) of the In 3d_5/2_ spectrum in Figure 2b might be attributed to this chemical environment. In summary, a comparison between non-rinsed and rinsed absorber surfaces suggests the presence of a Rb–In–Se environment,^4,52^ which is subsequently reduced (if not even fully removed) by the here-applied rinse.

Figure 3 shows the In 4d/Ga 3d spectral region. Corroborating our findings above, we observe a second Ga 3d component at ∼21.5 eV for the non-rinsed CIGSe. Again, based on literature values for other Ga-halogenides (GaCl_3_, GaBr_3_, and GaI_3_),^50^ we assign this peak to Ga–F bonds at the surface. This component is not present for the rinsed sample, in agreement with the removal of F discussed above from the surface. The In 4d spectrum in Figure 3 exhibits a clear intensity reduction at ∼18 eV for the rinsed absorber as compared to the non-rinsed absorber. The blue difference spectrum (“non-rinsed” – “rinsed”) highlights this reduction, in agreement with the intensity reduction on the high-binding-energy side of the In 3d_5/2_ XPS spectrum in Figure 2b. We attribute these additional spectral components to the In–O bonds. Figure 2b shows that the overall In 3d_5/2_ intensity increases significantly after the rinse. In contrast, the overall Ga 2p_3/2_ intensity decreases, leading to a decrease in the surface GGI ratio as a result of the rinse.

**Figure 4:**
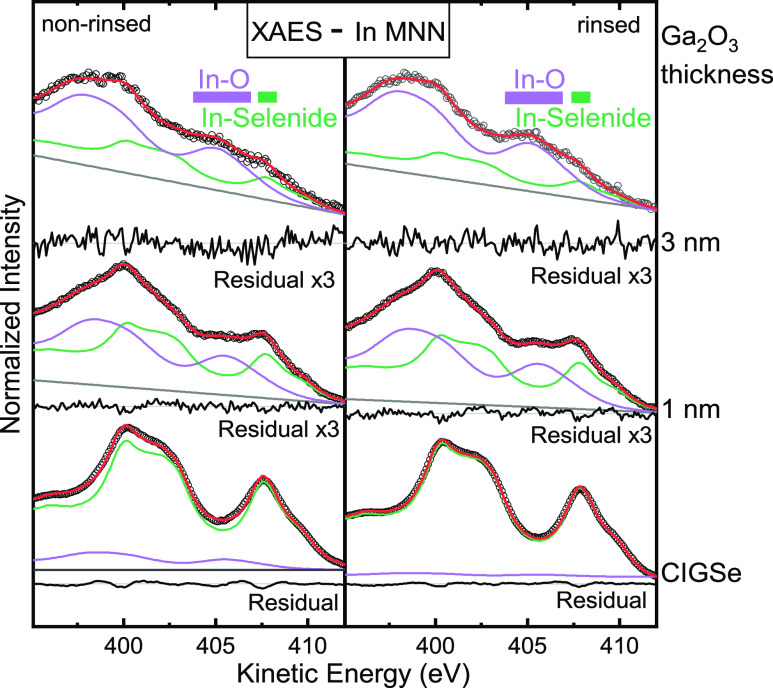
Mg K_α_-excited In M_4,5_N_4,5_N_4,5_ for the non-rinsed (left) and rinsed CIGSe (right) and Ga_2_O_3_/CIGSe samples with Ga_2_O_3_ thicknesses of 1 and 3 nm. Data points are represented as open black circles; individual species (fit components) are represented in green (In–Se bonds) and purple (In–O bonds), and the sum is presented in red. A linear background is shown in gray. Below each spectrum is given the residual. Literature values for the prominent M_4_N_5_N_5_ feature of different compounds^49^ are marked as colored bars.

**Figure 5:**
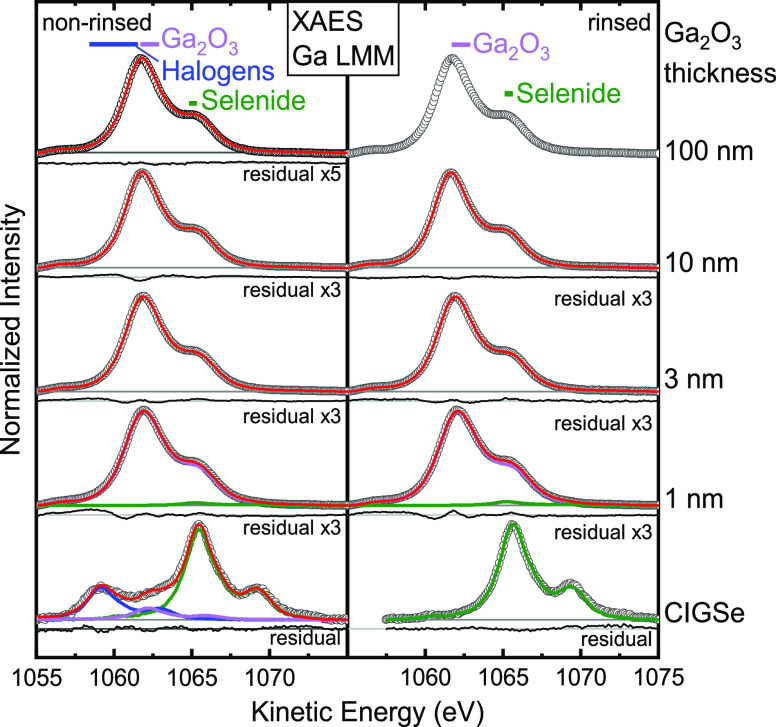
Mg K_α_-excited L_3_M_4,5_M_4,5_ XAES spectra of gallium for the non-rinsed and rinsed CIGSe and Ga_2_O_3_/CIGSe samples with increasing Ga_2_O_3_ thickness. Data are represented as open black circles, individual species (fit components) are represented in green (selenide), purple (gallium oxide), and blue (gallium fluoride), and the sum is represented in red. Below each spectrum, the residual is shown. Literature values for the prominent L_3_M_4,5_M_4,5,_ feature of different compounds^49,50^ are marked as colored bars.

To gain insights into the chemical composition of the Ga_2_O_3_ buffer layer and the formation of the Ga_2_O_3_/CIGSe interface, the XAES spectra of indium (M_4,5_N_4,5_N_4,5_, Figure 4) and gallium (L_3_M_4,5_M_4,5_, Figure 5) were investigated.

While the two In MNN (Figure 4) CIGSe absorber spectra look similar to published spectra,^14,47,54,55^ the 1 and 3 nm thick Ga_2_O_3_ spectra show additional intensity in the “valley” at 405 eV, and the overall spectral shape is broader. To analyze these spectral changes, all In MNN spectra were fitted by using two single-species In MNN spectra as fit functions (derived from an untreated ZSW absorber sample from another batch). All In MNN fits are thus composed of two components: one with In M_4_N_4,5_N_4,5_ at ∼407.7 eV, attributed to In in a CIGSe environment, and the other at 405–405.5 eV, attributed to In in an oxide environment. For the 1 and 3 nm Ga_2_O_3_/CIGSe samples, an additional Gaussian broadening was applied to the oxide component to describe the presence of several slightly varying chemical environments. The oxide component is weak in the non-rinsed absorber and further decreases by about four times in the rinsed absorber. For both the rinsed and non-rinsed 1 and 3 nm Ga_2_O_3_/CIGSe samples, a relative increase in the oxide component is observed, which dominates the 3 nm spectra. This finding indicates a significant influence of the sputter deposition of the Ga_2_O_3_ layer on the absorber surface, in particular, intermixing of absorber elements and the formation of In–O bonds.

In a similar fashion, the Ga L_3_M_4,5_M_4,5_ region is analyzed in Figure 5. A single-species Ga L_3_M_4,5_M_4,5_ spectrum (derived from the same untreated ZSW absorber sample mentioned above) was used as the fit function to describe our Ga L_3_M_4,5_M_4,5_ absorber spectra, while for the samples with a buffer layer, the measured Ga L_3_M_4,5_M_4,5_ spectrum of the 100 nm rinsed Ga_2_O_3_/CIGSe sample was used. This approach is necessary due to a significant broadening and change in the spectral shape of the Ga L_3_M_4,5_M_4,5_ spectra for various compounds.^50^ While the rinsed absorber is composed of one gallium component (main peak at 1065.5 eV), attributed to Ga in a CIGSe environment, the non-rinsed absorber exhibits two additional components. The second component at 1062 eV can be attributed to Ga in an oxide environment, and the third component at 1059 eV can be attributed to Ga in a fluoride environment. This interpretation is in agreement with the results shown in Figure 2a for the Ga 2p_3/2_ region.

The Ga LMM spectra of all of the Ga_2_O_3_/CIGSe samples are very similar. Only a very small absorber component is present for the 1 nm Ga_2_O_3_/CIGSe samples, in addition to the main component attributed to Ga in Ga_2_O_3_. The Ga in the CIGSe environment is not visible for thicker buffer layers. To determine the chemical environment of Ga, the modified Auger parameters (α’) for gallium and oxygen were calculated by adding the binding energies of the most prominent photoemission peaks (Ga 2p_3/2_ and O 1s) to the kinetic energies of the most prominent Auger peaks (Ga LMM and O KVV, respectively), where α’ = *E*_bin_ (photoemission) + *E*_kin_ (Auger). They are 2180.6 ± 0.2 eV for gallium and 1040.4 ± 0.2 eV for oxygen, respectively, independent of the buffer layer thickness. These values are close to the ones reported in the literature for gallium (2180.1–2180.4 eV) and oxygen (1040.7 eV) in Ga_2_O_3_,^49^ suggesting that the dominant species in the buffer layer is indeed Ga_2_O_3_, independent of the rinse, and that the chemical environment does not change with increasing thicknesses. Notably, we do not find any indication of the formation of gallium hydroxides, which would be indicated by an additional component in the O 1s spectra (Figure S2).

Now, we turn to the RbF-PDT-related elements and their evolution as a function of the Ga_2_O_3_ buffer layer thickness. First, Rb is investigated, specifically the Rb 2p_3/2_ peak with a binding energy of ∼1804 eV in our HAXPES spectra. A clear advantage of measuring this region is that there are no overlapping peaks, in contrast to the Rb 3d/Ga 3p region (as measured with XPS).

**Figure 6:**
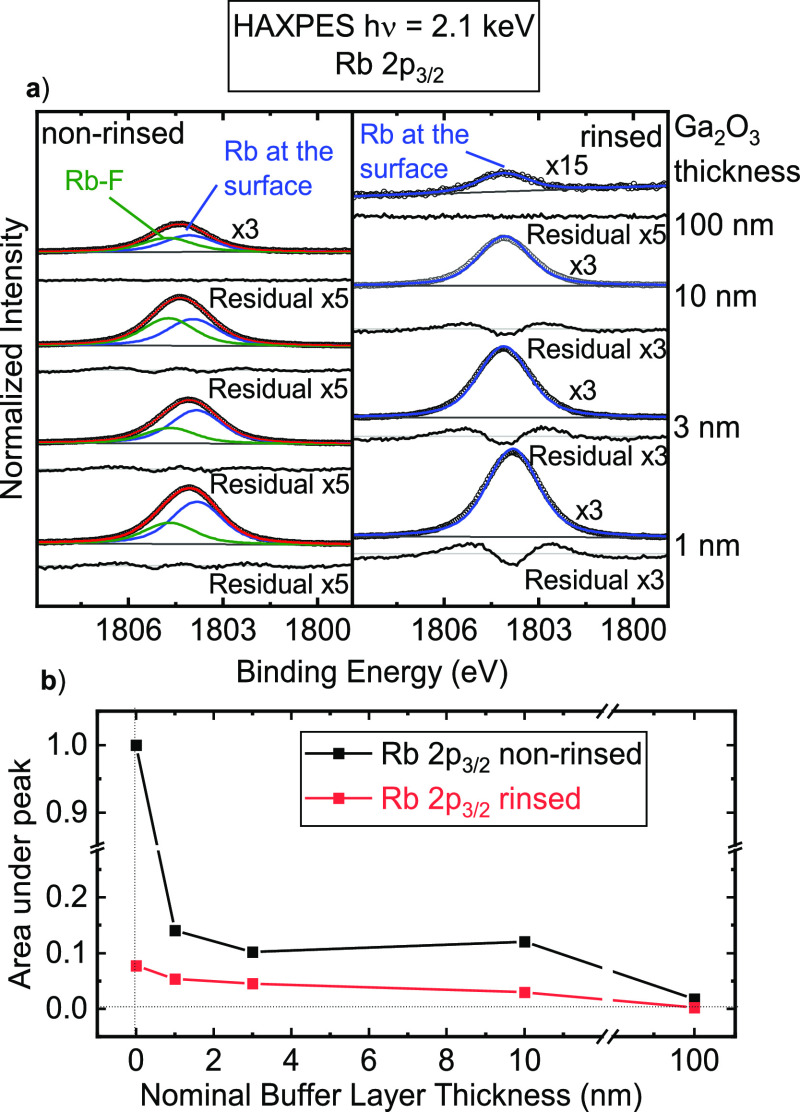
(a) Data and fits of the Rb 2p_3/2_ spectra of the non-rinsed (left) and rinsed (right) CIGSe samples with the Ga_2_O_3_ buffer layer, measured with HAXPES at an excitation energy of 2.1 keV. Data are represented as open black circles, individual species (fit components) are represented in green (Rb–F) and blue (Rb at the surface), and the sum is represented in red. Below each spectrum, the magnified (×3 or ×5) residual is colored in light gray. (b) Rb 2p_3/2_ peak area as a function of the nominal buffer layer thickness, normalized to the intensity of the non-rinsed absorber surface. Data for the non-rinsed and rinsed samples are shown in black and red, respectively.

Figure 6a shows the fit analysis of the Rb 2p_3/2_ peaks for the non-rinsed and rinsed samples with the Ga_2_O_3_ buffer layer, while Figure 6b shows the Rb 2p_3/2_ peak area as a function of the nominal buffer layer thickness.

Figure 6a demonstrates that a Rb 2p_3/2_ signal is visible on all sample surfaces. Note that these measurements are very surface-sensitive: at this excitation energy, the inelastic mean free path of the Rb 2p_3/2_ electrons is ∼0.8 nm; in contrast, it is ∼2.3 nm for the Rb 3d region. Combined with the complications arising from the spectral overlap with the Ga 3p lines, it is much harder to unequivocally discern the presence of Rb on the Ga_2_O_3_ buffer layer surfaces from the Rb 3d spectra. In the non-rinsed samples, Rb is mainly present in two different environments, as discussed in Figure 2c: Rb adsorbed on CIGSe and Rb–F, while in the rinsed samples, only adsorbed Rb is present.

Figure 6b shows an overall decrease in the area under the (total) Rb 2p_3/2_ peak with increasing buffer layer thickness. This decrease is much weaker than would be expected for (exponential) attenuation by the Ga_2_O_3_ overlayer. This discrepancy indicates a diffusion/segregation of Rb in both the rinsed and non-rinsed cases. In the rinsed case, the Rb intensity slowly but steadily decreases with buffer layer thickness. In contrast, the Rb 2p intensity in the non-rinsed series decreases sharply from the absorber to the 1 nm Ga_2_O_3_ sample, then stays approximately constant up to 10 nm Ga_2_O_3_, and then further strongly decreases from 10 to 100 nm. In addition, Figure 6a shows that the relative fraction of the Rb–F component increases with increasing buffer layer thickness in the non-rinsed sample series.

**Figure 7:**
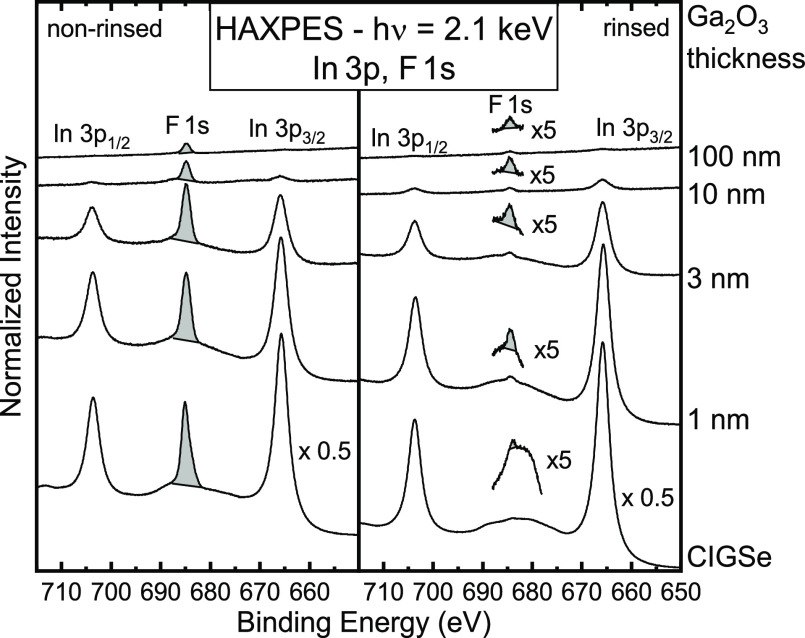
In 3p and F 1s regions for the non-rinsed (left) and rinsed (right) CIGSe and Ga_2_O_3_/CIGSe samples with increasing Ga_2_O_3_ thickness, measured with HAXPES at an excitation energy of 2.1 keV. To highlight the F 1s signal in the rinsed sample series, it is magnified by a factor of 5 and shown in blue above each spectrum.

To further investigate the Rb–F diffusion/ segregation, we now turn to the fluorine signals. Figure 7 shows the In 3p and F 1s spectral regions, measured with HAXPES. Although it is possible to observe the F 1s peak with XPS, the high signal-to-noise ratio achievable with HAXPES at the X-SPEC beamline helps to clearly detect the small F 1s peak between the (strong) In 3p peaks in the rinsed sample series.

In the non-rinsed samples, the F 1s peak decreases with increasing buffer layer thicknesses. There is still a clear F 1s peak for the 100 nm non-rinsed sample but no In 3p peaks, indicating a clear diffusion/segregation of F to the Ga_2_O_3_ buffer layer surface. After the rinse, no (or only a very small) F 1s peak is found, indicating that the rinse removes F from the absorber surface. In contrast, the rinsed Ga_2_O_3_/CIGSe samples all show a small F 1s peak, which slightly decreases in intensity as a function of the buffer layer thickness. As a possible explanation for the presence of F on the rinsed Ga_2_O_3_/CIGSe samples, some F might diffuse into the CIGSe absorber (e.g., along grain boundaries) during RbF-PDT and then diffuse to the Ga_2_O_3_/CIGSe interface during the sputter-deposition process (assisted by the elevated process temperature).

We speculate that the large amounts of Rb–F on the non-rinsed absorber and its diffusion into the Ga_2_O_3_ buffer layer could lead to the observed significantly lower solar-cell efficiencies. Rinsing the CIGSe absorber removes F, some Rb, and associated oxides and could hence result in optimized interface properties and higher efficiencies.

## Conclusions

We have presented a detailed investigation of the chemical structure of the RbF-treated CIGSe absorber surface, with and without an ammonia-based rinse, as well as their interfaces with a sputter-deposited Ga_2_O_3_ buffer layer. The rinse removes almost all F and most of the Rb in an Rb–F environment, while some remains at the CIGSe surface. The rinse removes In–O, Ga–O, and Ga–F bonds from the CIGSe surface, decreases the Ga/(Ga + In) ratio at the surface, and removes evidence of a Rb–In–Se bonding environment.

Rb and F are also found on all samples with a sputter-deposited Ga_2_O_3_ buffer layer, with and without rinse (in some cases, only trace amounts of F are observed). During sputter-deposition, a significant amount of In–O bonds is formed. The dominating chemical environment of the buffer layer is Ga_2_O_3_, independent of the buffer layer thickness and absorber rinsing.

Our findings thus indicate a rather complex chemical interface structure and diffusion/segregation behavior from the absorber to the buffer surface. This interface structure is substantially modified by applying a rinse, which then also leads to higher solar cell efficiencies. These significant changes in the chemical structure are expected to lead to changes in the electronic structure, in particular, the conduction band alignment, which are the subject of future investigations.

## Data Availability

The data that support the findings of this study are available from the corresponding authors upon reasonable request.

## Supporting Information Available

XPS survey spectra and O 1s, C 1s, and Na 1s XPS detailed spectra for the Ga_2_O_3_/CIGSe samples (PDF)

## References

[ref1] Chirilă A.; Reinhard P.; Pianezzi F.; Bloesch P.; Uhl A. R.; Fella C.; Kranz L.; Keller D.; Gretener C.; Hagendorfer H.; Jaeger D.; Erni R.; Nishiwaki S.; Buecheler S.; Tiwari A. N. Potassium-Induced Surface Modification of Cu(In,Ga)Se_2_ Thin Films for High-Efficiency Solar Cells. Nat. Mater. 2013, 12 (12), 1107–1111. 10.1038/nmat3789. 24185758

[ref2] Jackson P.; Wuerz R.; Hariskos D.; Lotter E.; Witte W.; Powalla M. Effects of Heavy Alkali Elements in Cu(In,Ga)Se_2_ Solar Cells with Efficiencies up to 22.6%. Phys. Status Solidi RRL 2016, 10 (8), 583–586. 10.1002/pssr.201600199.

[ref3] Nakamura M.; Yamaguchi K.; Kimoto Y.; Yasaki Y.; Kato T.; Sugimoto H. Cd-Free Cu(In,Ga)(Se,S)_2_ Thin-Film Solar Cell With Record Efficiency of 23.35%. IEEE J. Photovolt. 2019, 9 (6), 1863–1867. 10.1109/JPHOTOV.2019.2937218.

[ref4] Handick E.; Reinhard P.; Wilks R. G.; Pianezzi F.; Kunze T.; Kreikemeyer-Lorenzo D.; Weinhardt L.; Blum M.; Yang W.; Gorgoi M.; Ikenaga E.; Gerlach D.; Ueda S.; Yamashita Y.; Chikyow T.; Heske C.; Buecheler S.; Tiwari A. N.; Bär M. Formation of a K—In—Se Surface Species by NaF/KF Postdeposition Treatment of Cu(In,Ga)Se_2_ Thin-Film Solar Cell Absorbers. ACS Appl. Mater. Interfaces 2017, 9 (4), 3581–3589. 10.1021/acsami.6b11892. 28058843

[ref5] Keller J.; Aboulfadl H.; Stolt L.; Donzel-Gargand O.; Edoff M. Rubidium Fluoride Absorber Treatment for Wide-Gap (Ag,Cu)(In,Ga)Se_2_ Solar Cells. Sol. RRL 2022, 6 (6), 2200044 10.1002/solr.202200044.

[ref6] Reinhard P.; Bissig B.; Pianezzi F.; Hagendorfer H.; Sozzi G.; Menozzi R.; Gretener C.; Nishiwaki S.; Buecheler S.; Tiwari A. N. Alkali-Templated Surface Nanopatterning of Chalcogenide Thin Films: A Novel Approach Toward Solar Cells with Enhanced Efficiency. Nano Lett. 2015, 15 (5), 3334–3340. 10.1021/acs.nanolett.5b00584. 25844923

[ref7] Hedström J.; Ohlsen H.; Bodegard M.; Kylner A.; Stolt L.; Hariskos D.; Ruckh M.; Schock H.-W. ZnO/CdS/Cu(In,Ga)Se_2_ Thin Film Solar Cells with Improved Performance. Conference Record of the Twenty Third IEEE Photovoltaic Specialists Conference—1993 (Cat. No.93CH3283-9), 1993; pp 364–371.

[ref8] Scofield J. H.; Asher S.; Albin D.; Tuttle J.; Contreras M.; Niles D.; Reedy R.; Tennant A.; Noufi R. Sodium Diffusion, Selenization, and Microstructural Effects Associated with Various Molybdenum Back Contact Layers for CIS-Based Solar Cells. Proceedings of 1994 IEEE 1st World Conference on Photovoltaic Energy Conversion—WCPEC (A Joint Conference of PVSC, PVSEC and PSEC); IEEE: Waikoloa, HI, USA, 1994; Vol. 1, pp 164–167.

[ref9] Heske C.; Fink R.; Umbach E.; Riedl W.; Karg F. Na-induced Effects on the Electronic Structure and Composition of Cu(In,Ga)Se_2_ Thin-film Surfaces. Appl. Phys. Lett. 1996, 68 (24), 3431–3433. 10.1063/1.115783.

[ref10] Niles D. W.; Al-Jassim M.; Ramanathan K. Direct Observation of Na and O Impurities at Grain Surfaces of CuInSe_2_ Thin Films. J. Vac. Sci. Technol. Vac. Surf. Films 1999, 17 (1), 291–296. 10.1116/1.581583.

[ref11] Rockett A.; Granath K.; Asher S.; Al Jassim M. M.; Hasoon F.; Matson R.; Basol B.; Kapur V.; Britt J. S.; Gillespie T.; Marshall C. Na Incorporation in Mo and CuInSe_2_ from Production Processes. Sol. Energy Mater. Sol. Cells 1999, 59 (3), 255–264. 10.1016/S0927-0248(99)00026-4.

[ref12] Erfurth F. Elektronenspektroskopie an Cd-Freien Pufferschichten Und Deren Grenzflächen in Cu(In,Ga)(S,Se)_2_. Doctoral Thesis, Universität Würzburg, 2010.

[ref13] Kylner A. The Chemical Bath Deposited CdS/Cu(In,Ga)Se_2_ Interface as Revealed by X-Ray Photoelectron Spectroscopy. J. Electrochem. Soc. 1999, 146 (5), 1816–1823. 10.1149/1.1391849.

[ref14] Weinhardt L.; Fuchs O.; Groß D.; Umbach E.; Heske C.; Dhere N. G.; Kadam A. A.; Kulkarni S. S. Surface Modifications of Cu(In,Ga)S_2_ Thin Film Solar Cell Absorbers by KCN and H_2_O_2_/H_2_SO_4_ Treatments. J. Appl. Phys. 2006, 100 (2), 024907 10.1063/1.2216367.

[ref15] Jackson P.; Hariskos D.; Wuerz R.; Kiowski O.; Bauer A.; Friedlmeier T. M.; Powalla M. Properties of Cu(In,Ga)Se_2_ Solar Cells with New Record Efficiencies up to 21.7%. Phys. Status Solidi RRL 2015, 9 (1), 28–31. 10.1002/pssr.201409520.

[ref16] Ramanathan K.; Contreras M. A.; Perkins C. L.; Asher S.; Hasoon F. S.; Keane J.; Young D.; Romero M.; Metzger W.; Noufi R.; Ward J.; Duda A. Properties of 19.2% Efficiency ZnO/CdS/Cu(In,Ga)Se_2_ Thin-Film Solar Cells. Prog. Photovolt. Res. Appl. 2003, 11 (4), 225–230. 10.1002/pip.494.

[ref17] Kessler J.; Wennerberg J.; Bodegård M.; Stolt L. Highly Efficient Cu(In,Ga)Se_2_ Mini-Modules. Sol. Energy Mater. Sol. Cells 2003, 75 (1–2), 35–46. 10.1016/S0927-0248(02)00102-2.

[ref18] New world record for CIGS efficiency. Avancis. https://www.avancis.de/en/magazine/pr-efficiency (accessed 11 03, 2022).

[ref19] Hynes K. M.; Newham J. A Comparison of Window/Buffer Layer Materials for CdTe Thin Film Modules Using Environmental Risk Assessment. Conference Record of the Twenty-Eighth IEEE Photovoltaic Specialists Conference—2000 (Cat. No.00CH37036); 2000; pp 1513–1516.

[ref20] Hariskos D.; Spiering S.; Powalla M. Buffer Layers in Cu(In,Ga)Se_2_ Solar Cells and Modules. Thin Solid Films 2005, 480–481, 99–109. 10.1016/j.tsf.2004.11.118.

[ref21] Garud S.; Gampa N.; Allen T. G.; Kotipalli R.; Flandre D.; Batuk M.; Hadermann J.; Meuris M.; Poortmans J.; Smets A.; Vermang B. Surface Passivation of CIGS Solar Cells Using Gallium Oxide. Phys. Status Solidi A 2018, 215 (7), 1700826 10.1002/pssa.201700826.

[ref22] Larsson F.; Keller J.; Olsson J.; Donzel-Gargand O.; Martin N. M.; Edoff M.; Törndahl T. Amorphous Tin-Gallium Oxide Buffer Layers in (Ag,Cu)(In,Ga)Se_2_ Solar Cells. Sol. Energy Mater. Sol. Cells 2020, 215, 110647 10.1016/j.solmat.2020.110647.

[ref23] Koida T.; Kamikawa-Shimizu Y.; Yamada A.; Shibata H.; Niki S. Cu(In,Ga)Se_2_ Solar Cells With Amorphous Oxide Semiconducting Buffer Layers. IEEE J. Photovolt. 2015, 5 (3), 956–961. 10.1109/JPHOTOV.2015.2396356.

[ref24] Witte W.; Paetel S.; Menner R.; Bauer A.; Hariskos D. The Application of Sputtered Gallium Oxide as Buffer for Cu(In,Ga)Se_2_ Solar Cells. Phys. Status Solidi RRL 2021, 15 (9), 2100180 10.1002/pssr.202100180.

[ref25] Hariskos D.; Hempel W.; Menner R.; Witte W. Influence of Substrate Temperature during In_x_S_y_ Sputtering on Cu(In,Ga)Se_2_/Buffer Interface Properties and Solar Cell Performance. Appl. Sci. 2020, 10 (3), 1052 10.3390/app10031052.

[ref26] Soni P.; Raghuwanshi M.; Wuerz R.; Berghoff B.; Knoch J.; Raabe D.; Cojocaru-Mirédin O. Sputtering as a Viable Route for In_2_S_3_ Buffer Layer Deposition in High Efficiency Cu(In,Ga)Se_2_ Solar Cells. Energy Sci. Eng. 2019, 7 (2), 478–487. 10.1002/ese3.295.

[ref27] Hauschild D.; Mezher M.; Schnabel T.; Spiering S.; Kogler W.; Carter J.; Blum M.; Yang W.; Ahlswede E.; Heske C.; Weinhardt L. Intermixing at the In_x_S_y_/Cu_2_ZnSn(S,Se)_4_ Heterojunction and Its Impact on the Chemical and Electronic Interface Structure. ACS Appl. Energy Mater. 2019, 2 (6), 4098–4104. 10.1021/acsaem.9b00263.

[ref28] Yousfi E. B.; Asikainen T.; Pietu V.; Cowache P.; Powalla M.; Lincot D. Cadmium-Free Buffer Layers Deposited by Atomic Later Epitaxy for Copper Indium Diselenide Solar Cells. Thin Solid Films 2000, 361–362, 183–186. 10.1016/S0040-6090(99)00860-3.

[ref29] Mezher M.; Garris R.; Mansfield L. M.; Blum M.; Hauschild D.; Horsley K.; Duncan D. A.; Yang W.; Bär M.; Weinhardt L.; Ramanathan K.; Heske C. Soft X-Ray Spectroscopy of a Complex Heterojunction in High-Efficiency Thin-Film Photovoltaics: Intermixing and Zn Speciation at the Zn(O,S)/Cu(In,Ga)Se_2_ Interface. ACS Appl. Mater. Interfaces 2016, 8 (48), 33256–33263. 10.1021/acsami.6b09245. 27934158

[ref30] Minemoto T.; Hashimoto Y.; Satoh T.; Negami T.; Takakura H.; Hamakawa Y. Cu(In,Ga)Se_2_ Solar Cells with Controlled Conduction Band Offset of Window/Cu(In,Ga)Se_2_ Layers. J. Appl. Phys. 2001, 89 (12), 8327–8330. 10.1063/1.1366655.

[ref31] Nishimura T.; Chantana J.; Kawano Y.; Yamada A.; Kimoto Y.; Kato T.; Sugimoto H.; Minemoto T. Interfacial Modification Mechanism by Aging Effect for High-Performance Cd-Free and All-Dry Process Cu(In,Ga)(S,Se)_2_ Solar Cells. Appl. Phys. Lett. 2020, 117 (22), 223501 10.1063/5.0031241.

[ref32] Erfurth F.; Hußmann B.; Schöll A.; Reinert F.; Grimm A.; Lauermann I.; Bär M.; Niesen Th.; Palm J.; Visbeck S.; Weinhardt L.; Umbach E. Chemical Structure of the (Zn_1-x_Mg_x_)O/CuIn(S,Se)_2_ Interface in Thin Film Solar Cells. Appl. Phys. Lett. 2009, 95 (12), 122104 10.1063/1.3230071.

[ref33] Salomé P.; Keller J.; Törndahl T.; Teixeira J. P.; Nicoara N.; Andrade R.-R.; Stroppa D. G.; González J.; Edoff M.; Leitão J.; Sadewasser S. CdS and Zn_1-x_ Sn_x_O_y_ Buffer Layers for CIGS Solar Cells. Sol. Energy Mater. Sol. Cells 2017, 159, 272–281. 10.1016/j.solmat.2016.09.023.

[ref34] Lindahl J.; Wätjen J. T.; Hultqvist A.; Ericson T.; Edoff M.; Törndahl T. The Effect of Zn_1-x_ Sn_x_ O_y_ Buffer Layer Thickness in 18.0% Efficient Cd-Free Cu(In,Ga)Se_2_ Solar Cells. Prog. Photovolt. Res. Appl. 2013, 21 (8), 1588–1597. 10.1002/pip.2239.

[ref35] Löckinger J.; Nishiwaki S.; Bissig B.; Degutis G.; Romanyuk Y. E.; Buecheler S.; Tiwari A. N. The Use of HfO_2_ in a Point Contact Concept for Front Interface Passivation of Cu(In,Ga)Se_2_ Solar Cells. Sol. Energy Mater. Sol. Cells 2019, 195, 213–219. 10.1016/j.solmat.2019.03.009.

[ref36] Kotipalli R.; Vermang B.; Joel J.; Rajkumar R.; Edoff M.; Flandre D. Investigating the Electronic Properties of Al_2_O_3_/Cu(In,Ga)Se_2_ Interface. AIP Adv. 2015, 5 (10), 107101 10.1063/1.4932512.

[ref37] Werner F.; Veith-Wolf B.; Spindler C.; Barget M. R.; Babbe F.; Guillot J.; Schmidt J.; Siebentritt S. Oxidation as Key Mechanism for Efficient Interface Passivation in Cu(In,Ga)Se_2_ Thin-Film Solar Cells. Phys. Rev. Appl. 2020, 13 (5), 054004 10.1103/PhysRevApplied.13.054004.

[ref38] Heinemann M. D.; van Hest M. F. a. M.; Contreras M.; Perkins J. D.; Zakutayev A.; Kaufmann C. A.; Unold T.; Ginley D. S.; Berry J. J. Amorphous Oxides as Electron Transport Layers in Cu(In,Ga)Se_2_ Superstrate Devices. Phys. Status Solidi A 2017, 214 (5), 1600870 10.1002/pssa.201600870.

[ref39] Gallium Oxide: Materials Properties, Crystal Growth, and Devices; Springer Series in Materials Science; Higashiwaki M., Fujita S., Eds.; Springer International Publishing: Cham, 2020; Vol. 293.

[ref40] Onuma T.; Saito S.; Sasaki K.; Masui T.; Yamaguchi T.; Honda T.; Higashiwaki M. Valence Band Ordering in β-Ga_2_O_3_ Studied by Polarized Transmittance and Reflectance Spectroscopy. Jpn. J. Appl. Phys. 2015, 54 (11), 112601 10.7567/JJAP.54.112601.

[ref41] Tippins H. H. Optical Absorption and Photoconductivity in the Band Edge of β-Ga_2_O_3_. Phys. Rev. 1965, 140 (1A), A316–A319. 10.1103/PhysRev.140.A316.

[ref42] Orita M.; Ohta H.; Hirano M.; Hosono H. Deep-Ultraviolet Transparent Conductive β-Ga_2_O_3_ Thin Films. Appl. Phys. Lett. 2000, 77 (25), 4166–4168. 10.1063/1.1330559.

[ref43] He H.; Orlando R.; Blanco M. A.; Pandey R.; Amzallag E.; Baraille I.; Rérat M. First-Principles Study of the Structural, Electronic, and Optical Properties of Ga_2_O_3_ in Its Monoclinic and Hexagonal Phases. Phys. Rev. B: Condens. Matter Mater. Phys. 2006, 74 (19), 195123 10.1103/PhysRevB.74.195123.

[ref44] Witte W.; Hempel W.; Paetel S.; Menner R.; Hariskos D. Influence of Sputtered Gallium Oxide as Buffer or High-Resistive Layer on Performance of Cu(In,Ga)Se_2_-Based Solar Cells. J. Mater. Res. 2022, 37 (11), 1825–1834. 10.1557/s43578-022-00608-z.

[ref45] Gutzler R.; Witte W.; Kanevce A.; Hariskos D.; Paetel S. V_OC_-Losses across the Band Gap: Insights from a High-Throughput Inline Process for CIGS Solar Cells. Prog. Photovolt. Res. Appl. 2023, 31 (10), 1023–1031. 10.1002/pip.3707.

[ref46] Weinhardt L.; Steininger R.; Kreikemeyer-Lorenzo D.; Mangold S.; Hauschild D.; Batchelor D.; Spangenberg T.; Heske C. X-SPEC: A 70 eV to 15 keV Undulator Beamline for X-Ray and Electron Spectroscopies. J. Synchrotron Radiat. 2021, 28 (2), 609–617. 10.1107/S1600577520016318. 33650573 PMC7941287

[ref47] Moulder J. F.; Stickle W. F.; Sobol P. E.; Bomben K. D. Handbook of X-Ray Photoelectron Spectroscopy: A Reference Book of Standard Spectra for Identification and Interpretation of XPS Data; Perkin Elmer Cooperation: Minnesota, USA, 1992.

[ref48] Seah M. P. Post-1989 Calibration Energies for X-Ray Photoelectron Spectrometers and the 1990 Josephson Constant. Surf. Interface Anal. 1989, 14 (8), 488 10.1002/sia.740140813.

[ref49] NIST X-ray Photoelectron Spectroscopy (XPS) Database Main Search Menu. https://srdata.nist.gov/xps/main_search_menu.aspx (accessed 04 07, 2022).

[ref50] Bourque J. L.; Biesinger M. C.; Baines K. M. Chemical State Determination of Molecular Gallium Compounds Using XPS. Dalton Trans. 2016, 45 (18), 7678–7696. 10.1039/C6DT00771F. 27052931

[ref51] Bär M.; Repins I.; Contreras M. A.; Weinhardt L.; Noufi R.; Heske C. Chemical and Electronic Surface Structure of 20%-Efficient Cu(In,Ga)SeSe_2_ Thin Film Solar Cell Absorbers. Appl. Phys. Lett. 2009, 95 (5), 052106 10.1063/1.3194153.

[ref52] Bombsch J.; Avancini E.; Carron R.; Handick E.; Garcia-Diez R.; Hartmann C.; Félix R.; Ueda S.; Wilks R. G.; Bär M. NaF/RbF-Treated Cu(In,Ga)Se_2_ Thin-Film Solar Cell Absorbers: Distinct Surface Modifications Caused by Two Different Types of Rubidium Chemistry. ACS Appl. Mater. Interfaces 2020, 12 (31), 34941–34948. 10.1021/acsami.0c08794. 32633119

[ref53] Maticiuc N.; Kodalle T.; Lauche J.; Wenisch R.; Bertram T.; Kaufmann C. A.; Lauermann I. In Vacuo XPS Investigation of Cu(In,Ga)Se_2_ Surface after RbF Post-Deposition Treatment. Thin Solid Films 2018, 665, 143–147. 10.1016/j.tsf.2018.09.026.

[ref54] Kreikemeyer-Lorenzo D.; Hauschild D.; Jackson P.; Friedlmeier T. M.; Hariskos D.; Blum M.; Yang W.; Reinert F.; Powalla M.; Heske C.; Weinhardt L. Rubidium Fluoride Post-Deposition Treatment: Impact on the Chemical Structure of the Cu(In,Ga)Se_2_ Surface and CdS/Cu(In,Ga)Se_2_ Interface in Thin-Film Solar Cells. ACS Appl. Mater. Interfaces 2018, 10 (43), 37602–37608. 10.1021/acsami.8b10005. 30272438

[ref55] van Maris V. R.; Hauschild D.; Niesen T. P.; Eraerds P.; Dalibor T.; Palm J.; Blum M.; Yang W.; Heske C.; Weinhardt L. Impact of UV-Induced Ozone and Low-Energy Ar^+^-Ion Cleaning on the Chemical Structure of Cu(In,Ga)(S,Se)_2_ Absorber Surfaces. J. Appl. Phys. 2020, 128 (15), 155301 10.1063/5.0020253.

