# Supplementary material for: Rb Diffusion and Oxide Removal at the RbF-Treated Ga2O3/Cu(In,Ga)Se2 Interface in Thin-Film Solar Cells
Source: ACS Appl Mater Interfaces. 2023 Nov 1;15(45):53113–21. doi: 10.1021/acsami.3c11165 (PMC10659031; doi:10.1021/acsami.3c11165)
Supplement: Supplementary file 1 — am3c11165_si_001.pdf [file am3c11165_si_001.pdf]

Supporting Information
Rb Diffusion and Oxide Removal at the RbF-treated Ga_2O_3/Cu(In,Ga)Se_2 Interface in Thin-Film Solar Cells

Elizaveta Pyatenko[a,b,], Dirk Hauschild[b,c,d], Vladyslav Mikhnych[b,#], Raju Edla[b], Ralph Steininger[b], Dimitrios Hariskos[e], Wolfram Witte[e], Michael Powalla[e], Clemens Heske[b,c,d], and Lothar Weinhardt[b,c,d,*]*

[a] Laboratory for Applications of Synchrotron Radiation (LAS), Karlsruhe Institute of Technology (KIT), Kaiserstraße 12, 76131 Karlsruhe, Germany

[b] Institute for Photon Science and Synchrotron Radiation (IPS), Karlsruhe Institute of Technology (KIT), Hermann-v.-Helmholtz-Platz 1, 76344 Eggenstein-Leopoldshafen, Germany

[c] Institute for Chemical Technology and Polymer Chemistry (ITCP), Karlsruhe Institute of Technology (KIT), Engesserstraße 18/20, 76128 Karlsruhe, Germany

[d] Department of Chemistry and Biochemistry, University of Nevada, Las Vegas (UNLV), 4505 Maryland Parkway, Las Vegas, NV 89154-4003, United States

[e] Zentrum für Sonnenenergie- und Wasserstoff-Forschung Baden-Württemberg (ZSW), Meitnerstraße 1, 70563 Stuttgart, Germany

* Authors to whom correspondence should be addressed:

elizaveta.pyatenko@kit.edu, lothar.weinhardt@kit.edu

# current address: Kvitky-Osnovyanenko 3, 61003 Kharkiv, Ukraine

Figure S1 shows the Mg K_α-excited x-ray photoelectron spectroscopy (XPS) survey spectra of the CIGSe absorbers with RbF-PDT and the 1, 3, and 100 nm Ga_2O_3/CIGSe interface samples.

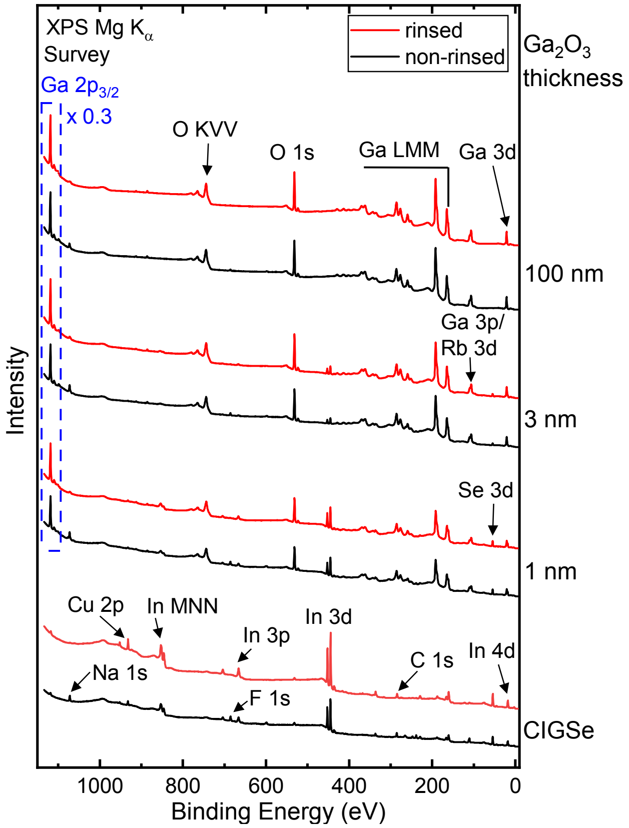

Figure S1. Mg K_{α} XPS survey spectra of the CIGSe absorbers with RbF-PDT and the 1, 3, and 100 nm Ga_2O_3/CIGSe interface samples. The red and black spectra correspond to the rinsed and non-rinsed sample series, respectively. The Ga $2p_{3/2}$ signals of the sputtered Ga_2O_3 buffer layers are multiplied by a factor of 0.3 for better visibility (blue dashed box). Prominent photoemission and Auger peaks are labeled.

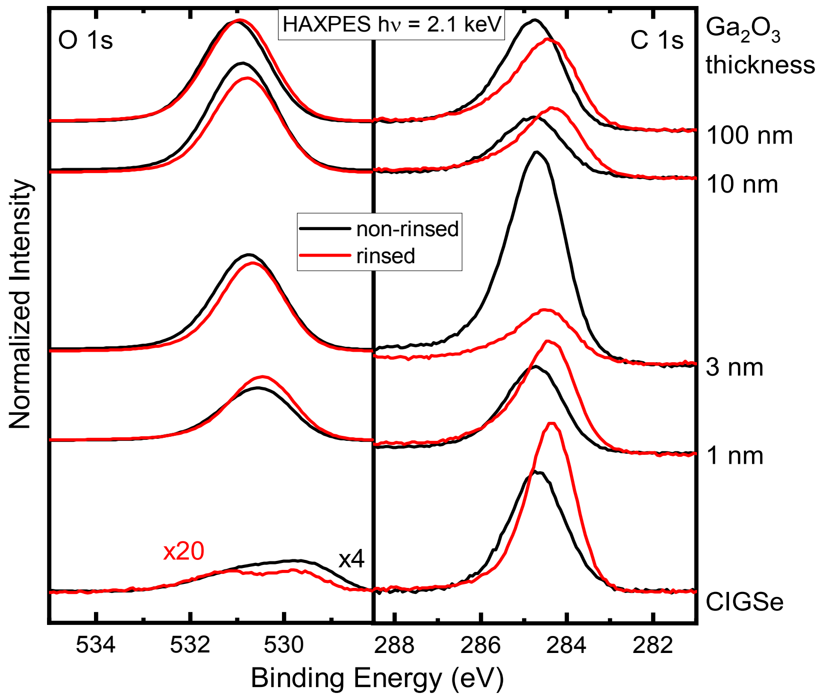

Figure S2. O 1s (left) and C 1s (right) core levels for the non-rinsed and rinsed CIGSe and Ga_2O_3/CIGSe samples with increasing Ga_2O_3 thickness, measured with HAXPES at an excitation energy of 2.1 keV. The red and black spectra correspond to the rinsed and non-rinsed sample series,

respectively. The O 1s spectra were magnified by a factor of 20 for the rinsed and by a factor of 4 for the non-rinsed CIGSe absorber samples, respectively.

Figure S2 shows the O and C 1s signals for the non-rinsed and rinsed sample series. In the O 1s spectra, the signals of both, rinsed and non-rinsed, CIGSe absorbers consist of two components, which indicates that oxygen is present in (at least) two different chemical environments. The presence of more than one oxygen species on CIGSe absorbers after RbF-PDT has been reported before as well[1]. The rinse removes a large part of the oxygen, with the signal decreasing by a factor of ~5 for the rinsed sample. Overall, the intensity of the oxygen signals on the absorber surfaces is very low (note the magnification factors of ×4 and ×20, and also see Figure 1 and Figure S1). The samples with Ga_2O_3 buffer layer all have the same O 1s peak shape, which increases in intensity and shifts towards higher binding energies for thicker buffer layers. The rinse reverses this effect slightly – all peaks are shifted to lower binding energy for the rinsed samples.

The C 1s signal in the HAXPES spectra varies strongly between rinsed and non-rinsed samples. We suggest that the amount of surface adsorbates present on a particular sample likely depends on the specific conditions under which it was prepared and handled. Again, the intensity of the carbon signals on the absorber surfaces is very low (see Figure 1 and Figure S1). We also observe a slight shift towards lower binding energies of the C 1s peak for the rinsed samples, compared to the non-rinsed samples.

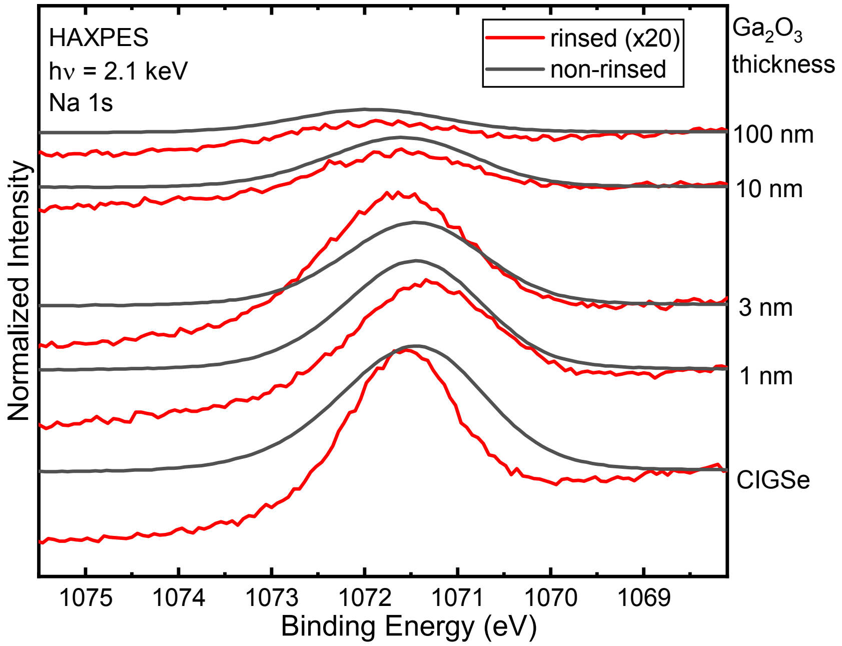

Figure S3. Na 1s peaks for the non-rinsed (black) and rinsed (red) CIGSe and Ga_2O_3/CIGSe samples with increasing Ga_2O_3 thickness, measured with HAXPES at an excitation energy of 2.1 keV. The rinsed spectra were magnified by a factor of 20.

Figure S3 shows the Na 1s signals for the non-rinsed and rinsed sample series. The Na 1s signal decreases by a factor of ~20 after the rinse. The peaks decrease with increasing buffer layer thickness and appear to exhibit a similar behavior for the non-rinsed and rinsed samples, although there are some differences depending on the specific sample and how it was prepared (such as the higher Na 1s peak in the 3 nm Ga_2O_3/CIGSe rinsed sample).

References

(1) Kreikemeyer-Lorenzo, D.; Hauschild, D.; Jackson, P.; Friedlmeier, T. M.; Hariskos, D.; Blum, M.; Yang, W.; Reinert, F.; Powalla, M.; Heske, C.; Weinhardt, L. Rubidium Fluoride Post-Deposition Treatment: Impact on the Chemical Structure of the $Cu(In,Ga)Se_2$ Surface and $CdS/Cu(In,Ga)Se_2$ Interface in Thin-Film Solar Cells. *ACS Appl. Mater. Interfaces* **2018**, *10* (43), 37602–37608. https://doi.org/10.1021/acsami.8b10005.
